# Curcumin piperidone derivatives induce caspase-dependent apoptosis and suppress miRNA-21 expression in LN-18 human glioblastoma cells

**DOI:** 10.1186/s41021-023-00297-y

**Published:** 2024-02-01

**Authors:** Nur Syahirah Che Razali, Kok Wai Lam, Nor Fadilah Rajab, A. Rahman A. Jamal, Nurul Farahana Kamaludin, Kok Meng Chan

**Affiliations:** 1 Center for Toxicology and Health Risk Studies, Faculty of Health Sciences, Universiti Kebangsaan Malaysia, Kuala Lumpur, 50300 Malaysia; 2 Centre for Drug and Herbal Development, Faculty of Pharmacy, Universiti Kebangsaan Malaysia, Kuala Lumpur, 50300 Malaysia; 3 Center for Health Ageing and Wellness Studies, Faculty of Health Sciences, Universiti Kebangsaan Malaysia, Kuala Lumpur, 50300 Malaysia; 4 UKM Medical Molecular Biology Institute, UKM Medical Centre, Cheras, 56000 Malaysia; 5 Product Stewardship and Toxicology, Group Health, Safety and Environment (GHSE), Petroliam Nasional Berhad (PETRONAS), Kuala Lumpur, 50088 Malaysia

**Keywords:** Glioblastoma, Curcuminoid analogue, Curcumin, Apoptosis, Caspase, MiRNA-21

## Abstract

**Background:**

Previously, we have reported on the two curcuminoid analogues with piperidone derivatives, namely FLDP-5 and FLDP-8 have more potent anti-proliferative and anti-migration effects than curcumin. In this study, we further investigated the mode of cell death and the mechanism involved in the cell death process induced by these analogues on human glioblastoma LN-18 cells.

**Results:**

The FLDP-5 and FLDP-8 curcuminoid analogues induced LN-18 cell death through apoptosis in a concentration-dependent manner following 24 h of treatment. These analogues induced apoptosis in LN-18 cells through significant loss of mitochondrial mass and mitochondrial membrane potential (MMP) as early as 1-hour of treatment. Interestingly, N-acetyl-l-cysteine (NAC) pretreatment did not abolish the apoptosis induced by these analogues, further confirming the cell death process is independent of ROS. However, the apoptosis induced by the analogues is caspases-dependent, whereby pan-caspase pretreatment inhibited the curcuminoid analogues-induced apoptosis. The apoptotic cell death progressed with the activation of both caspase-8 and caspase-9, which eventually led to the activation of caspase-3, as confirmed by immunoblotting. Moreover, the existing over-expression of miRNA-21 in LN-18 cells was suppressed following treatment with both analogues, which suggested the down-regulation of the miRNA-21 facilitates the cell death process.

**Conclusion:**

The FLDP-5 and FLDP-8 curcuminoid analogues downregulate the miRNA-21 expression and induce extrinsic and intrinsic apoptotic pathways in LN-18 cells.

## Introduction

Glioblastoma multiforme (GBM) is recognized as the most aggressive primary glial neoplasm [1, 2]. The treatment for GBM, either with surgery or radiation therapy, is only partially successful due to this disease’s high potential for infiltration and invasion [3, 4]. Research on the molecular mechanism underlying glioma tumorigenesis and potential novel chemotherapeutic agents towards gliomas has increased recently.

Chemotherapeutic drugs are important for cancer control and are used in most cases following surgical procedures as adjuvant treatment for patients with GBMs. Recent research has shown that curcumin has remarkable growth inhibition and apoptosis activation of glioblastoma and neuroblastoma cells in vitro, and decreased in vivo tumor growth [1]. Curcumin is a pigment extracted from turmeric which exhibits an antitumor effect in many cancer types. Due to its strong antitumor effect, the use of curcumin to treat gliomas has received considerable attention in recent years [2, 3]. Curcumin, however, has some drawbacks, such as low bioavailability, making it difficult for the body to absorb. Its propensity to a wide range of targets, poor potency and unsatisfactory pharmacokinetics restrict the clinical viability of this biologically potent natural product. As a result, studies focusing on the design of curcumin structural analogues to optimize specific chemotherapeutic properties have been carried out to synthesize and create a new synthetic curcuminoids that can overcome curcumin drawbacks [4, 5].

Hence, our group have synthesized two curcuminoid analogues with two piperidone derivatives, namely FLDP-5 and FLDP-8 [6]. Previously, our group has reported on the comparison of the effectiveness of these curcuminoid analogues against curcumin, whereby the curcuminoid analogues exhibited highly potent tumour-suppressive effects with anti-proliferative and anti-migratory activities on LN-18 human glioblastoma cells compared to curcumin. In this regard, we further investigate the molecular mechanism underlying the cell death process induced by FLDP-5 and FLDP-8 curcuminoid analogues against LN-18 cells compared to curcumin.

## Materials and methods

### Chemicals and reagents

Dulbecco’s Modified Eagle’s Medium (DMEM), penicillin/streptomycin and fetal bovine serum (FBS) from *PAA Laboratories*, Australia; phosphate buffer saline (PBS), Tetramethylrhodamine, ethyl ester (TMRE), nonyl acridine orange (NAO) from *Sigma-Aldrich*, UK; N-acetyl-l-cysteine (NAC) from *Nacalai Tesque Inc*., Kyoto, Japan; dimethyl sulfoxide (DMSO) and hydrochloric acid (HCl) form *Fisher Scientific*, UK; sodium hydrogen carbonate (NaHCO_3_) and potassium hydrogen phosphate (KH_2_PO_4_) from *Systerm*, Malaysia; ethanol and methanol from *HmbG Chemicals*, German; Annexin V-FITC and Annexin binding buffer (ABB) from *BD Biosciences*, USA; radio-immunoprecipitation assay (RIPA) buffer, dithiothreitol (DTT) from *Merck*, USA; protease and phosphatase inhibitors from *Roche*, Mannheim, Germany; pan-caspase (z-VAD-FMK) and Bradford Protein Quantification Assay kit from *Abcam*, Cambridge, UK; 2X Laemmli sample buffer, ECL chemiluminescence substrate from *Bio-Rad Laboratories*, Hercules, CA, USA; protein marker from *SMOBIO Technology*, Taiwan; anti-caspase-3, -8, -9 antibodies, anti-β-actin antibody, and secondary antibody anti-rabbit from *Cell Signalling Technologies*, USA; SNAP i.d. 2.0 protein detection system from *Millipore*, Billerica, MA; ; Qiagen MIReasy Mini kit from *Valencia*, Spain; TaqMan MicroRNA Reverse Transcription Kit, ethidium bromide (10 mg/mL), Taqman Universal PCR Master Mix dan Taqman MicroRNA Assays Primer from *Thermo Scientific*, USA.

### Cell culture

LN-18 human glioblastoma cells were obtained from American Type Culture Collection (ATCC). The culture medium used throughout these experiments was Dulbecco’s Modified Eagle’s Medium (DMEM) supplemented with 5% fetal bovine serum (FBS) and 1% penicillin/streptomycin. LN-18 human glioblastoma cells were used between passages 3–12 for all experiments and maintained at 37 °C and 5% CO_2_.

### Test compounds

Compounds 4-Peperidinone,3,5-bis[(4-hydroxy-3-methoxyphenyl)methylene]-,(3E,5E) (FLDP-5) and 4-Peperidinone,3,5-bis[(4-hydroxy-3-methoxyphenyl)methylene]-1-Methyl(3E,5E) (FLDP-8) were synthesized by Dr. Lam Kok Wai from the Centre for Drug and Herbal Development, Faculty of Pharmacy, Universiti Kebangsaan Malaysia (Kuala Lumpur, Malaysia) [6]. The synthesized method was done following the method from the previous study with slight modification [7]. Briefly, dry hydrogen chloride gas was passed into the reaction mixture containing appropriate benzaldehyde (2 mmol) and ketone (1 mmol) in ethanol (15 mL). The reaction mixture was stirred at room temperature, and the reaction progress was monitored by thin-layer chromatography (TLC). After completion, the resulting solid was collected, filtered and crystallized to form ethanol to afford the target compounds. The purity of both FLDP-5 and FLDP-8 were > 98% as analyzed using nuclear magnetic resonance (NMR).

#### Compound 1

3,5-bis(4-hydroxy-3-methoxybenzylidene)piperidin-4-one (FLDP-5).

Yellow powder, Yield 32%; m.p. 176–178 °C; ^1^H NMR (500 MHz, DMSO-d_6_) δ: 9.93 (s, 2 H), 7.80 (s, 2 H), 7.12 (s, 2 H), 7.01–6.92 (m, 4 H), 4.49 (s, 4 H), 3.83 (s, 6 H). δ: ^13^C NMR (126 MHz, DMSO-d_6_) δ 182.3, 149.6, 148.1, 139.9, 125.6, 125.16, 125.10, 116.3, 115.7, 56.1, 44.3. ESI-HRMS: C_21_H_21_NO_5_ mass calculated [M + H]^+^ 368.1498 found 368.1498.

#### Compound 2

3,5-bis(4-hydroxy-3-methoxybenzylidene)-1-methylpiperidin-4-one (FLDP-8).

Brown powder, Yield 10%; m.p. 181–183ºC; ^1^H NMR (500 MHz, DMSO-d_6_) δ: 9.95 (s, 2 H), 7.81 (s, 2 H), 7.13 (s, 2 H), 6.98 (d, J = 8.0 Hz, 4 H), 4.63 (s, 4 H), 3.84 (s, 6 H), 2.99 (s, 3 H). ^13^C NMR (126 MHz, DMSO-d_6_) δ: 182.3, 149.6, 148.1, 139.9, 125.6, 125.18, 125.10, 116.2, 115.7, 56.1, 44.3. ESI-HRMS: C_22_H_23_NO_5_ mass calculated [M + H]^+^ 382.1654 found 382.1655.

### Stock preparation

Curcumin and hydroquinone were purchased from *Sigma-Aldrich* (St. Louis, MO, USA). Stock solutions of FLDP-5 and hydroquinone (HQ) were prepared at 50 mM, while stock solutions for FLDP-8 and curcumin were prepared at 25 mM. HQ was used as positive control in this study. All compounds were dissolved in the solvent DMSO. All compounds on LN-18 cells were treated in dark conditions due to the compounds’ photosensitive characteristics. The DMSO concentration in the curcuminoid analogues treatment was < 0.1% v/v, whereas, for the curcumin treatment, the DMSO treatment was ≤ 0.4% v/v.

### Annexin V-FITC/PI assay

The mode of cell death was assessed as previously described [8, 9]. The death mode was analyzed based on the externalization of phosphatidylserine (PS) using the double staining method Annexin V-FITC/PI whereby Annexin V-FITC will recognize apoptotic cells through conjugation with PS as PS flipped to the outer leaf membrane of the cell during apoptosis. Necrotic cells were stained with propidium iodide (PI) as it entered the cells when the membrane integrity was severely compromised [10, 11]. Briefly, LN-18 cells were seeded at 5 × 10^4^ cells per well inside a 6-well plate with a volume of 2 mL for 24-h. Then, 2 mL of curcuminoid analogues (FLDP-5 and FLDP-8) and curcumin at concentrations of 0.625–10 µM and 6.25–100 µM were treated respectively to the cells before incubating for 24-h. After 24-h incubation, treated cell were collected and centrifuged at 220 × g for 5 min. The supernatant was removed, and the pellet was washed with ice-cold PBS and recentrifuged. The supernatant was discarded and the pellet was resuspended with 150 µL of Annexin V binding buffer (ABB). Subsequently, the cell suspension was transferred to flow cytometric analysis tubes and were stained with 2.5 µL of Annexin V-FITC before being incubated on ice in the dark for 15-min. Then, 5 µL of 50 µg/mL PI was added for the remaining 3 min. Lastly, 300 µL of ABB was added to the sample. The tubes were kept on the ice until flow cytometric analysis was performed using FACSCanto II flow cytometer (*BD Bioscience*, USA) on 10,000 cells.

### Role of oxidative stress and caspase in inducing cell death via N-acetyl-l-cysteine (NAC) and pan-caspase (z-VAD-FMK) pretreatment

To determine the role of intracellular oxidative stress and caspase in the cell death process induced by curcuminoid analogues and curcumin, NAC and z-VAD-FMK were used. LN-18 cells were pre-treated with 10 mM of NAC and 50 µM of z-VAD-FMK respectively for 1-h prior to treatment with the compounds. After 1-h, the cells were then treated with respective compounds and further incubated for 24-h. The treated cells were harvested and handled as described for the Annexin V-FITC/PI assay. Flow cytometric analysis was performed using FACSCanto II flow cytometer (*BD Bioscience*, USA) on 10,000 cells.

### Mitochondrial membrane potential (δψm) and mitochondrial mass assessment

The mitochondrial membrane potential and mass levels were determined as previously described [12, 13]. Briefly, the treated LN-18 cells were administered at different time-point intervals before being harvested. The treated LN-18 cells were then collected by centrifugation at 220 × g for 5 min. After the supernatant was discarded, the pellet was resuspended with 1 mL of fresh pre-warmed FBS-free DMEM media and with the addition of 1 µL of 50 µM tetramethylrhodamine ester (TMRE) and 5 mM nonyl acridine orange (NAO) dyes. The stained cells were incubated in dark at 37 °C for 15 min. After incubation, the cells were centrifuged at 220 × g for 5 min. Then, the cells were washed with 1 mL of chilled PBS, and the supernatant was discarded, followed by resuspension of the pellet with 500 µL of ice-cold PBS. The stained cell suspension were transferred to flow cytometric analysis tubes and analyzed using FACSCanto II flow cytometer (BD Bioscience, USA) on 10,000 cells.

### Immunoblotting analysis

The immunoblotting analysis was carried out as previously described [14]. Briefly, the LN-18 cells were treated with curcumin and curcuminoid analogues at various time periods (30 min – 24-h). The treated cells then were lysed in RIPA buffer containing 1 mM DTT, protease and phosphatase inhibitors. Protein concentrations were determined using the Bradford protein quantification assay kit. The lysates were then solubilized in 2× Laemmli sample buffer and denatured at 95 °C for 5 min. Then, 20 µg of each sample was subjected to 12% SDS–polyacrylamide gel electrophoresis (SDS-PAGE) and transferred onto a polyvinylidene fluoride (PVDF) membrane. Before immunoblotting, the protein marker was loaded along with the samples for SDS-PAGE. Membranes were blocked with Tris-buffered saline plus Tween-20 containing 5% bovine serum albumin for 1.5 h before the blots were incubated with primary antibodies overnight at 4 ◦C: anti-caspase-3 antibody (diluted 1:1000), anti-caspase-9 antibody (diluted 1:1000), anti-caspase-8 antibody (diluted 1:1000), and anti-beta-actin antibody (dilution 1:6000). Following that, the membranes then were incubated with anti-rabbit IgG, horseradish peroxidase (HRP)-conjugated secondary antibody (diluted 1:1000) using SNAP i.d. 2.0 protein detection system for 20 min. The blots were stained with ECL chemiluminescence substrate and visualised using Fusion-FX7 gel documentation (*Vilber Lourmat, Collegien*, France) for enhanced chemiluminescent detection. The signal intensity was quantified relative to the loading control (beta-actin) by performing densitometry using Fusion-Capt Advance software *(Vilber Lourmat*, Collegien, France).

### RNA isolation and quantitative RT-PCR

In this study, we investigated the expression miRNA-21 following curcuminoid analogues and curcumin treatments using quantitative RT-PCR (qPCR). Briefly, after 24-h of treatment, the treated cells were harvested and washed with PBS twice before extraction. Total RNA, including miRNA, was purified manually with miRNeasy Mini Kit (Qiagen) according to the manufacturer’s instructions. RNA integrity was evaluated by NanoDrop ND-1000 spectrophotometer (*Thermo Fisher Scientific*, USA). Real time (RT) reaction was performed using TaqMan MicroRNA Reverse Transcription Kit and miRNA-specific primers according to the manufacturer’s protocol. Briefly, the total RNA was transcribed into cDNA which was used as template for miRNA qRT-PCR analysis. Reverse-transcription reaction was conducted in 15 µL solution containing the mixture of 0.15 µL 100 mM dNTPs, 1 µL of MultiScribe™ Reverse Transcriptase, 1.5 µL 10× Reverse Transcription buffer, 0.19 µL RNase inhibitor, 4.16 µL of nuclease-free water, 3 µL of primer and 5 µL of total microRNA extracted from cells (10 ng). The mixture was then transferred to thermal cycler and reverse transcription were performed at 16 °C for 30 min, 42 °C for 30 min, 85 °C for 5 min and a 4 °C holding period. After that, the real-time PCR reaction was performed in a final volume of 10 µL comprising 0.5 µL of TaqMan MicroRNA Assay (20×), 3.84 µL of RNase-free water, 0.665 µL of cDNA (product from RT reaction) and 5 µL of TaqMan 2× Universal PCR Master Mix. The real-time PCR protocols were conducted as followed: 95 °C for 10 min; 40 cycles of 95 °C for 15 s and 60 °C for 60 s using the CFX96 real-time detection system (*Bio-Rad Laboratories*, Hercules, CA, USA). The relative expression levels of miRNA were normalized by housekeeping miRNA genes, U6 and GAPDH. The 2 − ΔΔCT method was used to determine the gene relative expression compared to the control.

### Statistical analysis

The data are expressed as the mean ± standard error of mean (SEM) from at least three independent experiments. The statistical significance was evaluated using one-way ANOVA with the Dunnet post hoc test to assess significance difference with negative control (NEG) or the Tukey post hoc test to determine the significance of differences between multiple treatment groups. Differences were considered statistically significant with a probability level of *p* < 0.05.

## Results

### FLDP-5 and FLDP-8 curcuminoid analogues induced apoptosis in LN-18 cells in a concentration-dependent manner

**Fig. 1:**
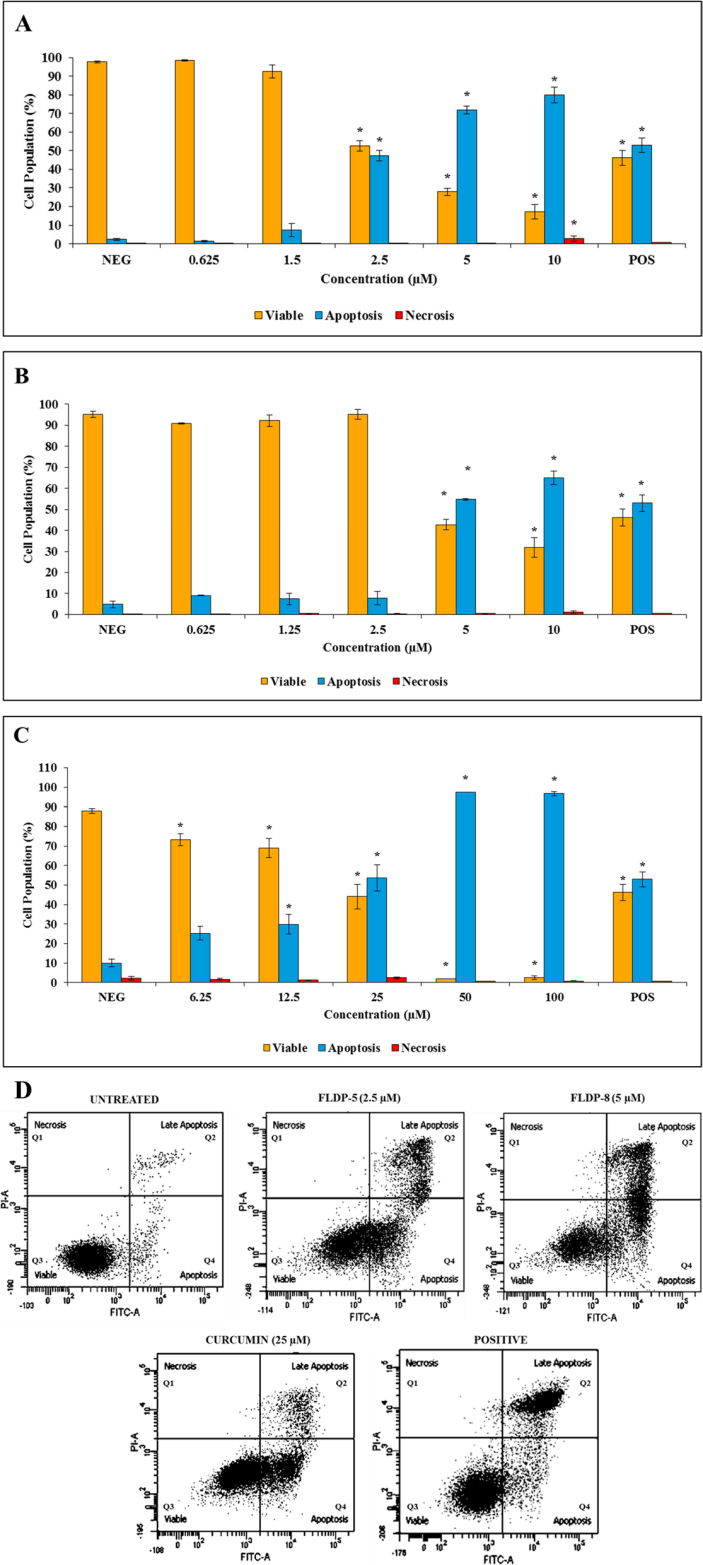
The apoptosis assessment of curcuminoid analogues (FLDP-5 and FLDP-8) and curcumin on LN-18 cells. **A** FLDP-5 curcuminoid analogue was treated on LN-18 cells with concentrations from 0.625 µM to 10 µM and was observed after 24-h treatment. **B** FLDP-8 curcuminoid analogue was treated on LN-18 cells with concentrations from 0.625 µM to 10 µM and was observed after 24-h treatment. **C** Curcumin was treated on LN-18 cells with concentrations from 6.25 µM to 100 µM and was observed after 24-h treatment. **D** Cytograms of curcuminoid analogues (FLDP-5 and FLDP-8) and curcumin at a concentration that induced ± 50% of apoptosis on LN-18 cells after 24-h. The cytograms represent the typical profile of three independent experiments. Each data point was obtained from three independent experimental replicates and expressed as mean ± SEM of the percentage of cells. *p < 0.05 against negative control, NEG (untreated cell)

The flow cytometric assessment of the apoptogenic effects of curcuminoid analogues and curcumin against LN-18 cells were determined using Annexin V-FITC/PI after 24-h treatment and are shown in Fig. 1. Cumulative data demonstrated that curcuminoid analogues, FLDP-5 and FLDP-8, including curcumin, induced cytotoxicity of LN-18 cells through apoptosis in a concentration-dependent manner with the increase of apoptotic cells from the lowest (0.625 µM – curcuminoid analogues; 6.25 µM – curcumin) to the highest (10 µM – curcuminoid analogues; 100 µM – curcumin) concentration of all the compounds treatment (Fig. 1). Curcuminoid analogues (FLDP-5 and FLDP-8) and curcumin caused significant increases of ± 50% of apoptosis cells in LN-18 cells,which were 47.37% ± 2.9 at 2.5 µM concentration for FLDP-5 curcuminoid analogue (Fig. 1A), 54.37% ± 0.37 at 5 µM concentration for FLDP-8 curcuminoid analogue (Fig. 1B) and 53.57% ± 6.74 at 25 µM concentration for curcumin (Fig. 1C) respectively. Therefore, these concentrations were used in subsequent experiments. The populations of apoptotic cells in cytograms of all compounds-induced apoptosis also are shown in Fig. 1. HQ treatment was used at 12.5 µM as the positive control (POS).

### Inhibition of ROS production through NAC pretreatment revealed that ROS functions appeared not to be required for these analogues-induced apoptosis in LN-18 cells

**Fig. 2:**
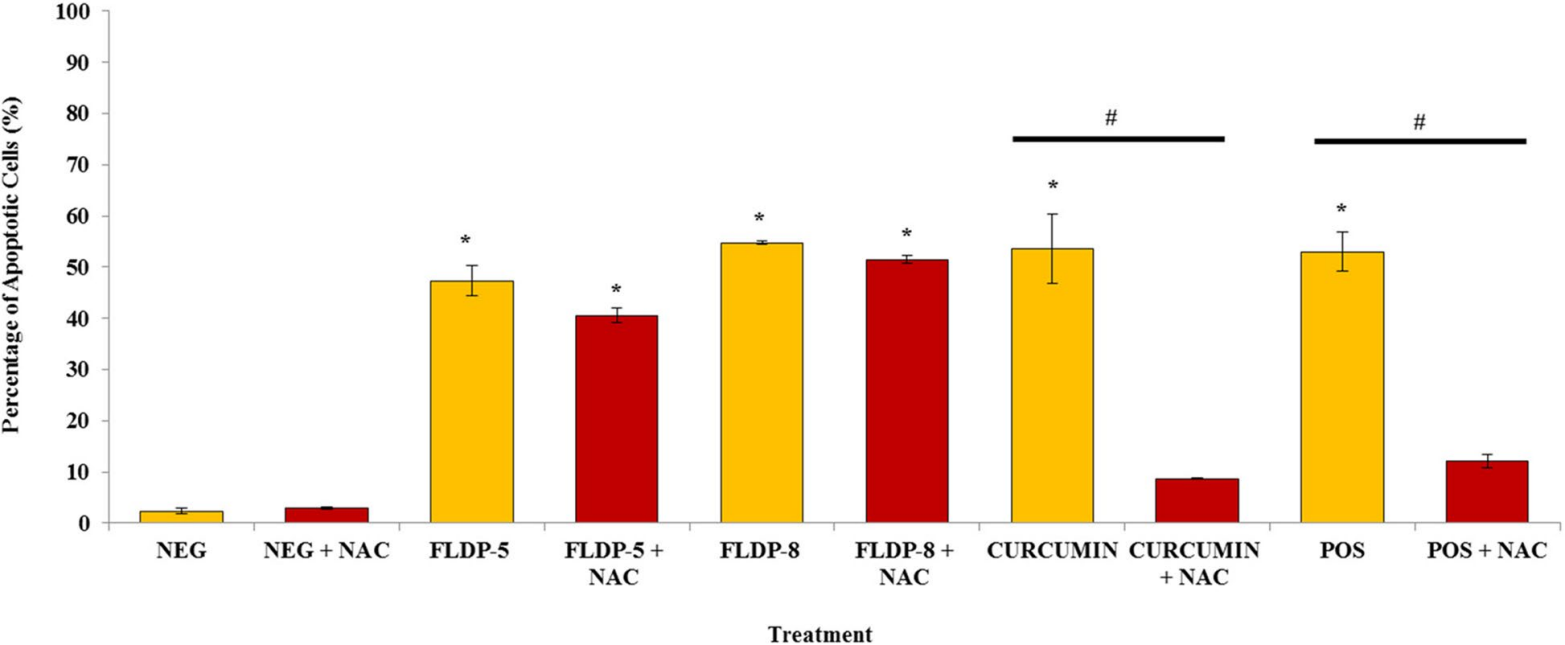
Assessment of the effects of 1-h NAC pretreatment in curcuminoid analogues (FLDP-5 and FLDP-8) and curcumin treated LN-18 cells. ROS functions appeared not to be required for curcuminoid analogues-induced apoptosis differed from curcumin-induced apoptosis that was demonstrated to be inhibited after NAC pretreatment in LN-18 cells. Each data point was obtained from three independent experimental replicates and expressed as mean ± SEM of the percentage of apoptotic cells. *p < 0.05 against negative control, NEG (untreated cell) and #*p* < 0.05 against treatment group without NAC

In order to understand the role of ROS in curcuminoid analogues-treated LN-18 cells, the cells were pretreated with NAC, an antioxidant that mimics the effects of the natural antioxidants and is known to inhibit ROS-dependent apoptosis, prior to the analogues and curcumin treatment. Interestingly, curcuminoid analogues-treated LN-18 cells showed no significant inhibition in the induction of apoptosis, suggesting the cell death process continued to happen even though the ROS production had been blocked. Figure 2 demonstrated the percentage of apoptotic cells with and without pretreatment of 10 mM NAC of all the compounds’ treatments in which FLDP-5 curcuminoid analogue displayed no significant decrease from 47.37% ± 2.9 to 40.53% ± 1.44. A similar result was observed in FLDP-8 curcuminoid analogue-treated cells with no significant decrease in the percentage of apoptotic cells from 54.73% ± 0.37 to 51.5% ± 0.75. On the other hand, curcumin-induced apoptosis was significantly inhibited by NAC pretreatment compared with curcumin-only treated cells, with a 0.16-fold decrease in the percentage of apoptotic cells from 53.57% ± 6.74 to 8.7% ± 0.1.

### Curcuminoid analogues (FLDP-5 and FLDP-8) caused early loss of cardiolipin and mitochondrial membrane potential (MMP) (ΔΨm) in LN-18 cells

**Fig. 3:**
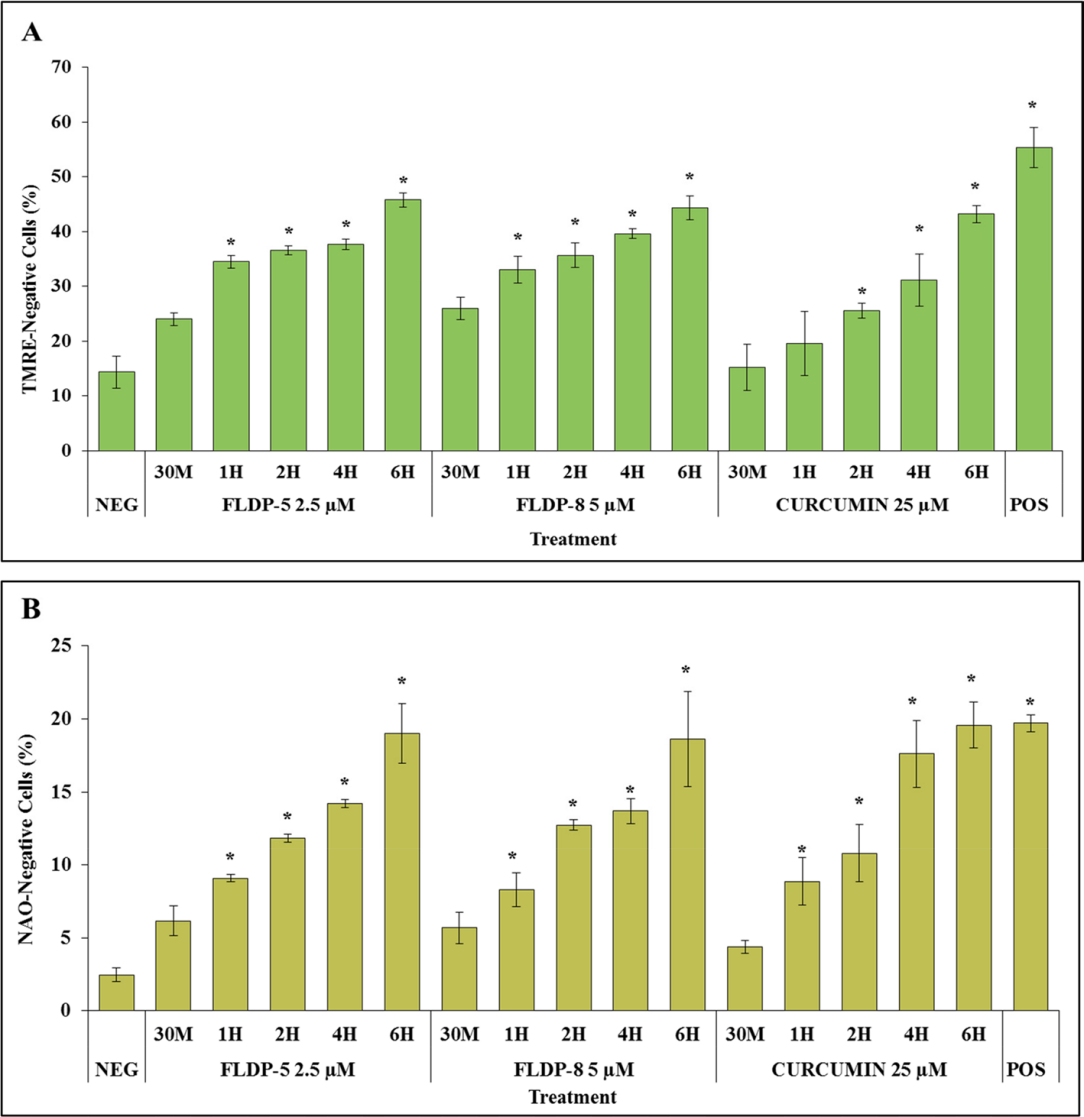
Assessment of MMP and cardiolipin levels in LN-18 cells. **A** Flow cytometric analysis of MMP level using TMRE staining. **B** Flow cytometric analysis of cardiolipin level using NAO staining. Cells were treated respectively with IC_50_ values of curcuminoid analogues (FLDP-5 and FLDP-8) and curcumin at different time-points ranging from 30 min to 6-h. Both assays used HQ treatment at 12.5 µM for 6-h as a positive control (POS). Each data point was obtained from three independent experimental replicates and expressed as mean ± SEM of TMRE- or NAO-negative cells (%). * *p* < 0.05 against negative control, NEG (untreated cell)

To further elucidate the mechanism underlying the curcuminoid analogues-induced apoptosis in LN-18 cells, flow cytometric assessments with respective TMRE and NAO dye staining were conducted to examine the involvement of mitochondria in the apoptosis pathway. The depolarization of the mitochondrial membrane potential (MMP) results in the release of apoptogenic factors and loss of oxidative phosphorylation due to the opening of the mitochondrial permeability transitional pore [15]. The MMP was detected using TMRE in this study, a potentiometric fluorescent dye which will accumulate along with the high potential level in mitochondria. Functional mitochondria will harbor high levels of MMP, and mitochondrotoxic agents can induce an early loss of MMP [16]. Our results demonstrated that FLDP-5 and FLDP-8 curcuminoid analogues, including curcumin, induced the loss of MMP in a time-dependent manner from 30 min until 6-h treatment in LN-18 treated cells (Fig. 3A). Significant MMP loss induced by FLDP-5 and FLDP-8 curcuminoid analogues were observed with 2.4-fold and 2.3-fold increase with the accumulation of TMRE-negative cells (indicating cells that lost the TMRE fluorescence) at 34.47% ± 1.67 and 33% ± 2.42 respectively compared to the untreated group (NEG) at 14.3% ± 2.95 as early as 1-h time point treatment and persisted until 6-h time point treatment. Curcumin appeared to induce MMP loss a bit later at 2-h time point treatment which also persisted until 6-h with a significant 1.85-fold increase of 25.5% ± 1.38 accumulated TMRE-negative cells.

To further establish the role of mitochondria in inducing curcuminoid analogues apoptosis, cardiolipin levels were assessed through flow cytometric analysis using NAO staining. The high-affinity binding of NAO to cardiolipin has been used to measure mitochondrial mass per cell and to quantify the level of cardiolipin in the inner and outer leaflets of the mitochondrial inner membrane [17]. Our results depicted that curcuminoid analogues (FLDP-5 and FLDP-8), and curcumin caused significant cardiolipin loss at 1-h time point treatment as indicated by 3.7-fold, 3.4-fold and 3.6-fold increase of NAO-negative cells from 2.47% ± 0.46 in control cells to 9.1% ± 0.26, 8.3% ± 1.15 and 8.87% ± 1.62 respectively in the treated cells (Fig. 3B).

### Pretreatment of pan-caspase (z-VAD-FMK) demonstrated that curcuminoid analogues-induced apoptosis in LN-18 cells were dependent on the caspase activation

**Fig. 4:**
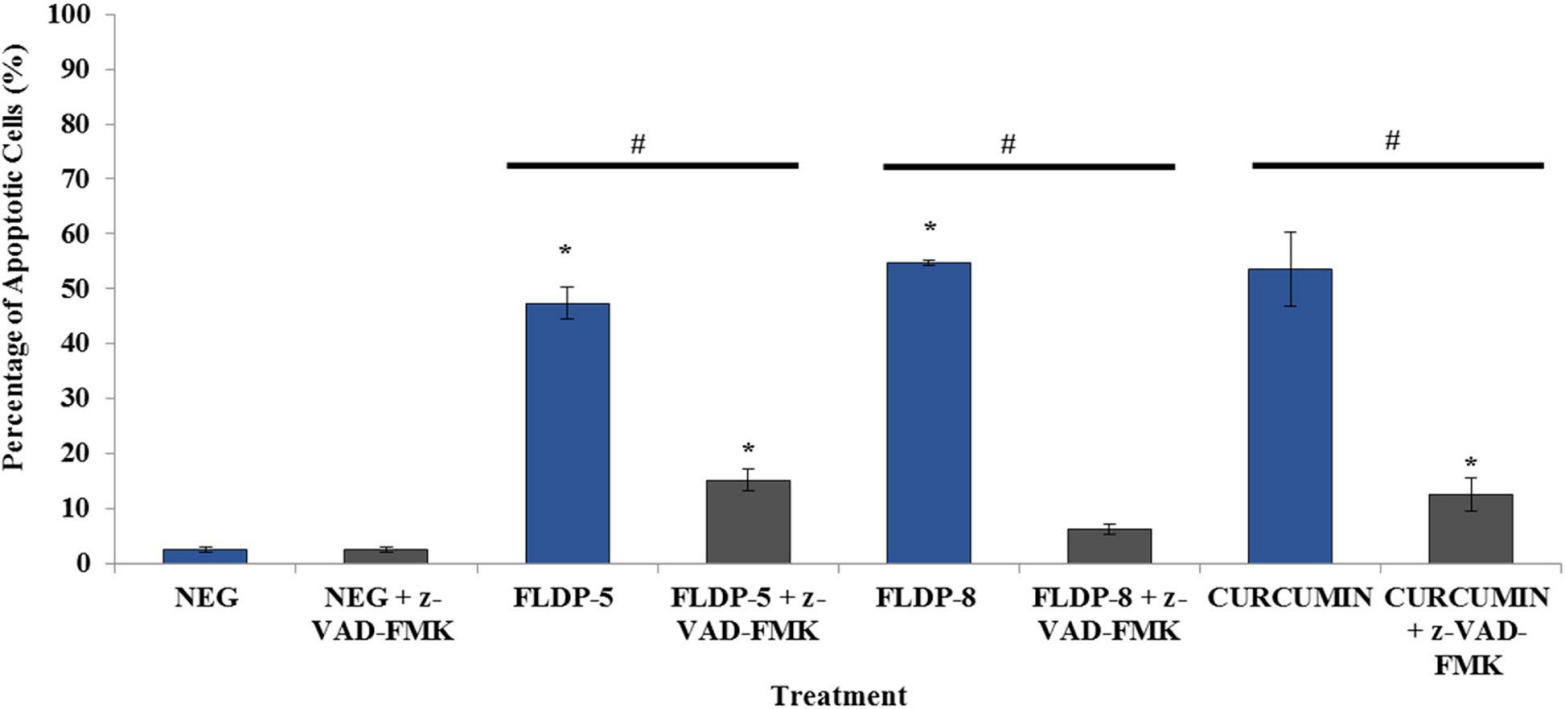
Assessment of the effects of 1-h z-VAD-FMK pretreatment in curcuminoid analogues (FLDP-5 and FLDP-8) and curcumin treated LN-18 cells. Results from 1-h z-VAD-FMK pretreatment in curcuminoid analogues and curcumin-induced apoptosis in LN-18 cells demonstrated the pan-caspase inhibitor significantly suppressed apoptosis. Each data point was obtained from three independent experimental replicates and expressed as mean ± SEM of the percentage of apoptotic cells. *p < 0.05 against negative control, NEG (untreated cell) and #*p* < 0.05 against the treatment group without z-VAD-FMK

The mechanism underlying the cell death process in LN-18 treated curcuminoid analogues was further assessed through investigation of the involvement of caspases. To understand the role of caspases in curcuminoid analogues-treated LN-18, cells were pretreated with z-VAD-FMK, a pan-caspase inhibitor, prior to curcuminoid analogues and curcumin treatments. Figure 4 illustrates the percentage of apoptotic cells with and without pretreatment of 50 µM z-VAD-FMK of all the compounds’ treatments. The results showed that curcuminoid analogues (FLDP-5 and FLDP-8) and curcumin-induced apoptosis were significantly inhibited by pretreatment with z-VAD-FMK with 0.32-fold, 0.12-fold and 0.23-fold decrease in the percentage of apoptotic cells from 47.37% ± 2.9 to 15.2% ± 2.11, 54.73% ± 0.37 to 6.3% ± 1.2, and 53.57% ± 6.74 to 12.5% ± 3.1 respectively (Fig. 4).

### FLDP-5 and FLDP-8 curcuminoid analogues induced extrinsic and intrinsic apoptosis pathways through the activation of caspases-8, -9, and − 3 in LN-18 cells

**Fig. 5:**
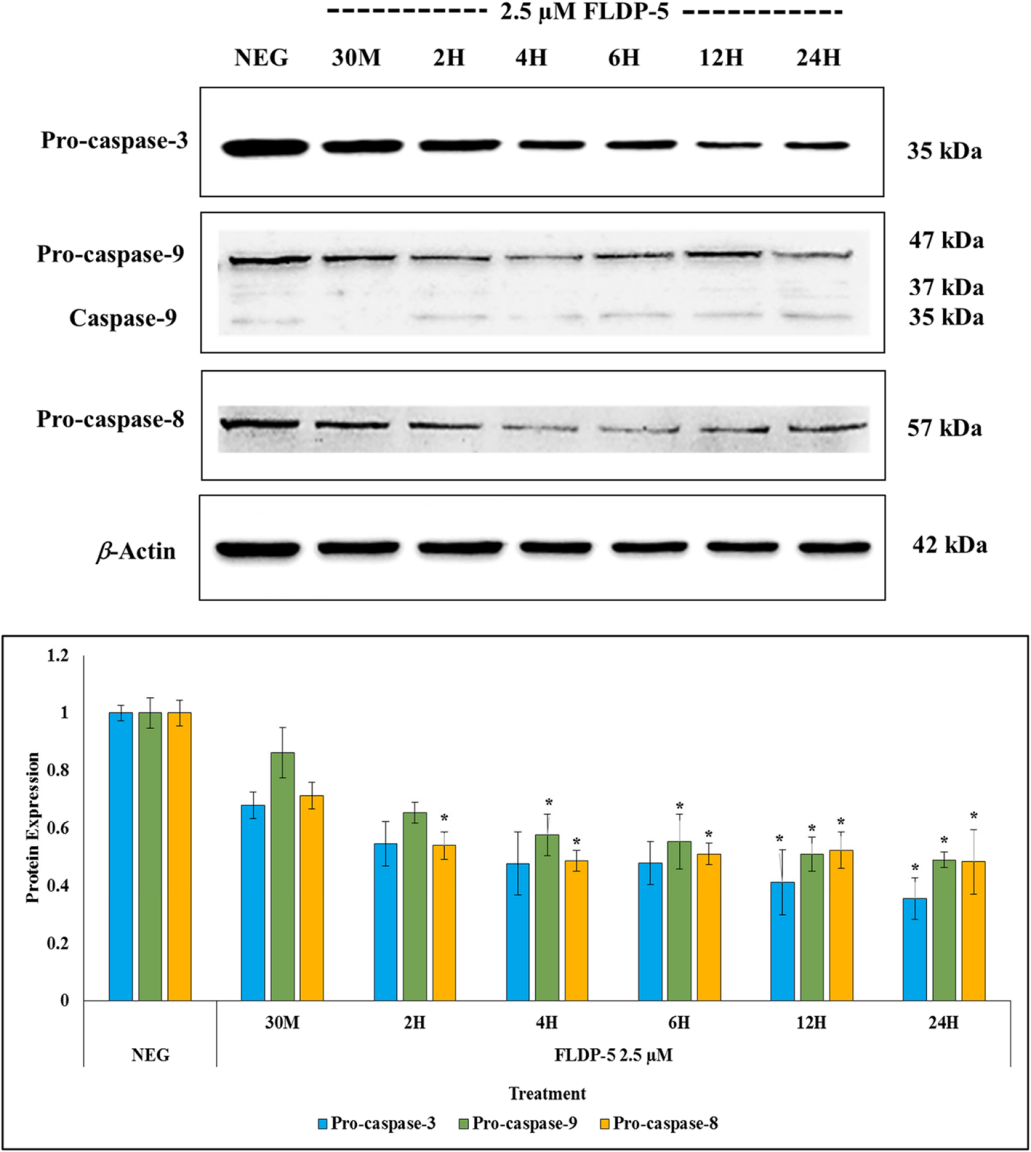
Effects of FLDP-5 curcuminoid analogue on pro-caspases-3, -8, and − 9 in LN-18 cells. Cells were treated with IC_50_ value of FLDP-5 curcuminoid analogue at different time-points ranging from 30 min until 24-h. The pro-caspases’ expressions were assessed using immunoblotting analysis. Each data point was obtained from three independent experimental replicates and expressed as mean ± SEM of protein expression. *p < 0.05 against negative control, NEG (untreated cell)

**Fig. 6:**
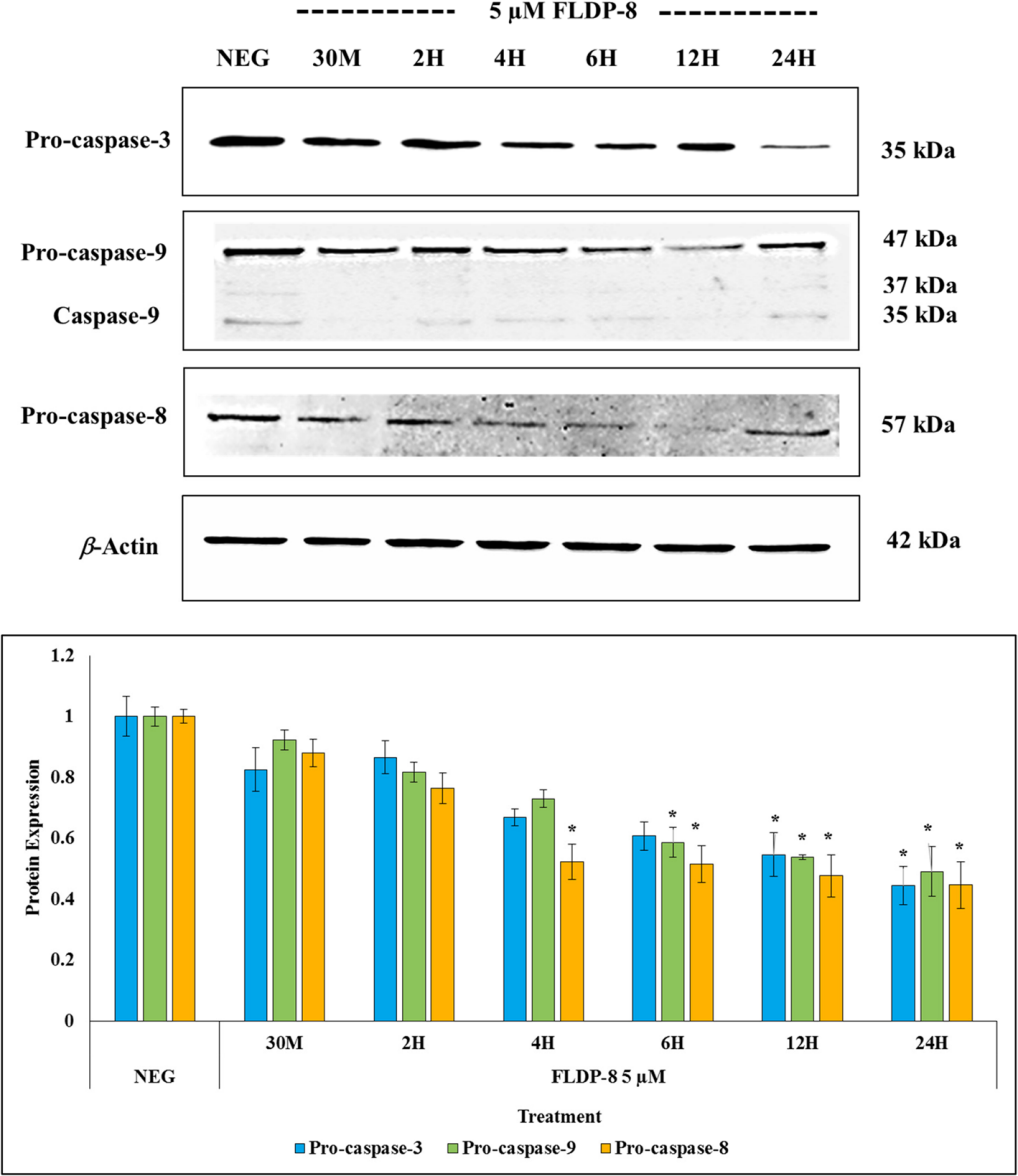
Effects of FLDP-8 curcuminoid analogue on pro-caspases-3, -8, and − 9 in LN-18 cells. Cells were treated with IC_50_ value of FLDP-8 curcuminoid analogue at different time-points ranging from 30 min until 24-h. The pro-caspases’ expressions were assessed using immunoblotting analysis. Each data point was obtained from three independent experimental replicates and expressed as mean ± SEM of protein expression. *p < 0.05 against negative control, NEG (untreated cell)

**Fig. 7:**
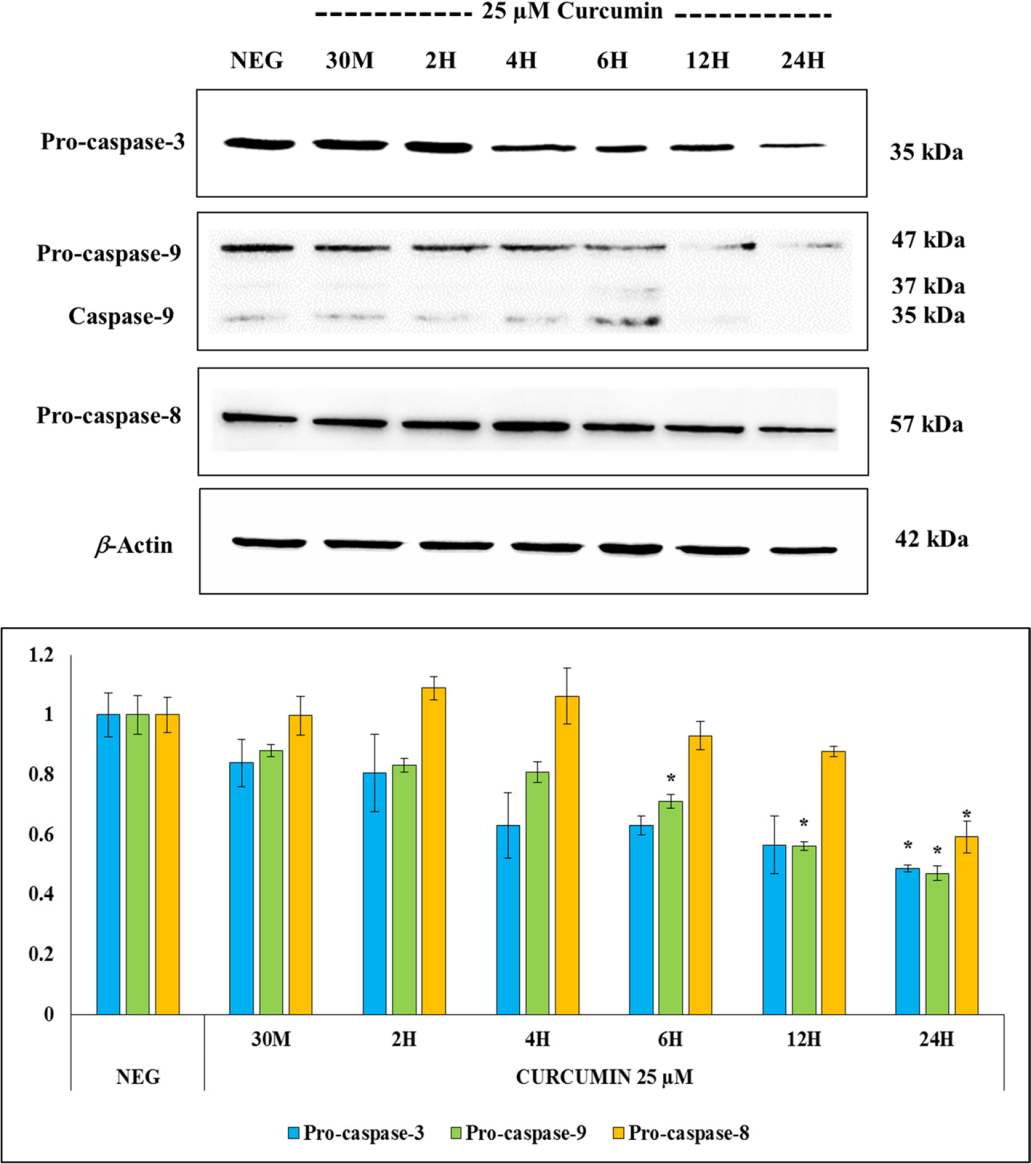
Effects of curcumin on pro-caspases-3, -8, and − 9 in LN-18 cells. Cells were treated with IC_50_ value of curcumin at different time-points ranging from 30 min until 24-h. The pro-caspases’ expressions were assessed using immunoblotting analysis. Each data point was obtained from three independent experimental replicates and expressed as mean ± SEM of protein expression. *p < 0.05 against negative control, NEG (untreated cell)

The caspases’ involvement through the caspase cascade activation is vital in activating apoptotic cell death. Therefore, the involvement of initiator and executor caspases, particularly caspases − 3, -8 and − 9, were further investigated through immunoblotting to clarify the mechanism of curcuminoid analogues-induced apoptosis in LN-18 cells. We firstly assessed the activity of executor caspase-3, and our results demonstrated that curcuminoid analogues (FLDP-5 and FLDP-8) and curcumin treatments in LN-18 cells caused a gradual decrease of pro-caspase-3 expression in time-dependent manner (Figs. 5, 6 and 7). Significant decreases in pro-caspase-3 expression (35 kDa) were observed in both FLDP-5 and FLDP-8 curcuminoid analogues at 12-h time point as indicated by the decrease of pro-caspase-3 protein expression from 1.00 ± 0.03 to 0.411 ± 0.11 and 1.00 ± 0.06 to 0.55 ± 0.07 respectively. In contrast, a significant decrease of curcumin-treated LN-18 cells was observed after 24-h treatment in the decrease of pro-caspase-3 expression from 1.00 ± 0.07 to 0.49 ± 0.01.

We further scrutinized the expression of initiator caspases − 8 and − 9 to find out if these analogues could induce either extrinsic or intrinsic apoptosis pathways. Interestingly, both FLDP-5 and FLDP-8 analogues significantly decreased the pro-caspase-8 and pro-caspase-9, suggesting the activation of both extrinsic and intrinsic apoptosis pathways. FLDP-5 curcuminoid analogue showed higher potential through earlier loss of pro-caspase-8 and pro-caspase-9 expressions at 2-h and 4-h as indicated by the decrease of both pro-caspase-8 and pro-caspase-9 expressions from 1.00 ± 0.05 to 0.54 ± 0.11 and 1.00 ± 0.05 to 0.58 ± 0.07 respectively (Figs. 5 and 6). Meanwhile, the FLDP-8 curcuminoid analogue significantly decreased the pro-caspase-8 and pro-caspase-9 expressions activating both pathways at slightly later time points at 4-h and 6-h. Both pro-caspase expressions were demonstrated by the decrease in the protein expressions from 1.00 ± 0.02 to 0.52 ± 0.05 and 1.00 ± 0.03 to 0.59 ± 0.05, respectively (Figs. 5 and 6).

On the other hand, curcumin-induced apoptosis was suggested only to activate the intrinsic pathway as stipulated through a significant decrease in pro-caspase-9 expression at 6-h time point as indicated by the decrease from 1.00 ± 0.06 to 0.71 ± 0.02 (Fig. 7). There was no significant decrease in pro-caspase-8 expression from 30 min up to 12-h, suggesting that curcumin-induced apoptosis did not involve caspase-8 activation. The significant decrease at 24-h treatment with the decrease of pro-caspase-8 expression from 1.00 ± 0.06 to 0.59 ± 0.01 was also suggested to happen probably due to activation of caspase-8 by other caspases.

### miRNA-21 expression in LN-18 cells was suppressed following treatment of FLDP-5 and FLDP-8 curcuminoid analogues

**Fig. 8:**
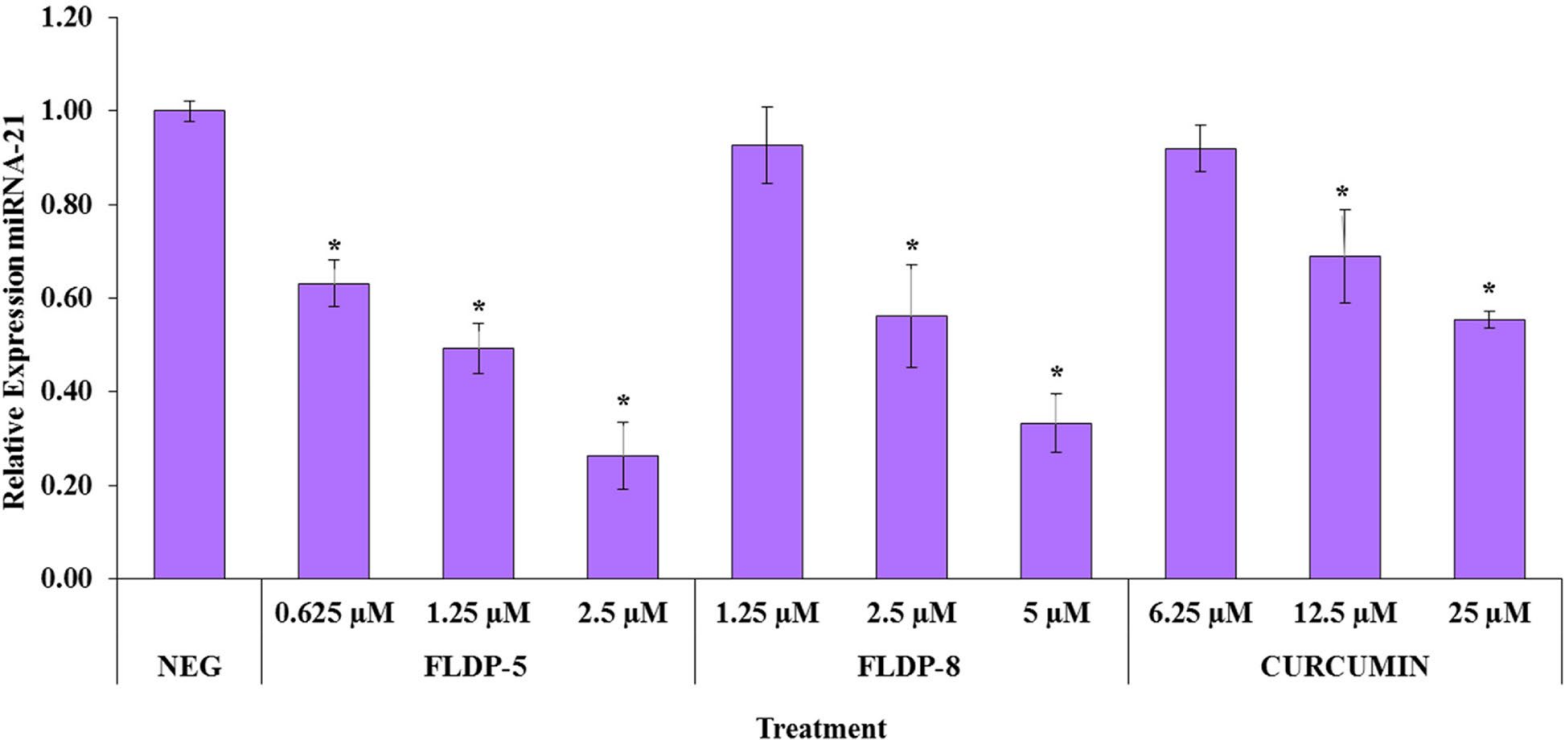
Assessment of the effects of curcuminoid analogues (FLDP-5 and FLDP-8) and curcumin on expression of miRNA-21 in LN-18 cells. Cells were treated respectively with a series of concentrations of curcuminoid analogues (FLDP-5 and FLDP-8) and curcumin for 24-h. Each data point was obtained from three independent experimental replicates and expressed as mean ± SEM of TMRE- or NAO-negative cells (%). * *p* < 0.05 against negative control, NEG (untreated cell)

miRNA-21 is highly upregulated in glioblastoma, and its expression levels are strongly associated with tumor grade and prognosis [18]. In regards to this, we aimed to assess the expression level of this particular miRNA after curcuminoid analogues treatment. Our RT-PCR results showed that curcuminoid analogues (FLDP-5 and FLDP-8) and curcumin induced a concentration-dependent decrease in the relative expression level of miRNA-21 in LN-18 treated cells. A significant decrease in the lowest concentration of FLDP-5 curcuminoid analogue (0.625 µM) could be observed as indicated by the decrease of relative expression miRNA-21 from 1.00 ± 0.02 to 0.63 ± 0.05. On the other hand, a significant decrease could be observed in the second-highest concentration of FLDP-8 curcuminoid analogue (2.5 µM) and curcumin (12.5 µM) as indicated by the decrease of relative expression of miRNA-21 from 1.00 ± 0.02 to 0.63 ± 0.05 and 0.69 ± 0.10 respectively (Fig. 8).

## Discussion

GBM is the most common primary malignant brain tumour. Available treatments, including surgery, radiation, and chemotherapy with the alkylating agent temozolomide (TMZ), are only partially successful. Despite receiving these therapies, most GBM patients have poor prognosis [19]. Therefore, new treatment strategies are required to treat these gliomas. Our previous study has reported that FLDP-5 and FLDP-8 curcuminoid analogues gave higher cytotoxicity towards LN-18 human GBM cells with increased production of ROS and DNA damage compared to curcumin treatment [6]. In this regard, we aimed to investigate further the mode of cell death and its underlying mechanism induced by these analogues.

The FLDP-5 and FLDP-8 curcuminoid analogues induce apoptosis death mode in LN-18 cells in a concentration-dependent manner and are more potent compared to curcumin. This finding corroborates numerous studies showing the anti-cancer potential of curcumin in inducing apoptosis in various cancer cell lines [20–24]. These findings were also in line with our results as FLDP-5 and FLDP-8 curcuminoid analogues were able to induce apoptosis death mode in LN-18 cells depending on concentration gradient and with much lesser concentration needed showing their potential compared to curcumin. Apoptosis is preferable during chemotherapy as it removes potentially harmful cells without causing extensive inflammatory-associated damage in the body [25].

We previously described that these analogues could induce the generation of ROS, leading to cell death in LN-18 cells [6]. Thus, we further analyzed the ROS role by inhibiting ROS production through NAC pretreatment. Curcumin has been reported in multiple studies to have a ROS-dependent pathway in inducing apoptosis in GBM cell lines [26–28], which is also in agreement with our study, as when LN-18 cells were pretreated with NAC to prevent ROS induction, apoptotic cells were significantly reduced compared to curcumin-only treated cells. However, interestingly, the depletion of ROS by NAC pretreatment did not abrogate the apoptotic cells induced by FLDP-5 and FDLP-8 curcuminoid analogues. ROS-independent cell apoptosis is relatively uncommon and rarely reported in cells [29, 30]. But in this case, regardless of ROS absence, curcuminoid analogues-induced apoptosis in LN-18 cells continues to occur, indicating that ROS is not the main apoptotic signal in the cell death process of LN-18 cells induced by these analogues. The reasons for these differences are yet to be uncovered, but they may reflect the fact that maybe ROS is not necessary for all apoptotic events induced by the different curcuminoids analogues.

We theorized that these curcumin analogues may induce apoptosis of LN-18 cells by causing an early increase of DNA damage and later directly affecting the mitochondria, causing mitochondrial dysfunction. Thus, we further investigated the mitochondrial condition by assessing mitochondrial membrane potential and mitochondrial mass. Results of our study showed a time-dependent decline in MMP and cardiolipin levels, and these events happened as soon as 1-h of treatment, indicating the early damage of mitochondria resulting from the gradual increase, although there was no significant DNA damage from 30 min until 1-h of treatments in a previous report [6]. When mitochondria are damaged, there is an increase in proton leakage that leads to diminished MMP. Moreover, due to the relevant role of cardiolipin in the functioning of the respiratory chain, we found a close correlation between a decreased cardiolipin, impaired mitochondrial functionality, and collapsed mitochondrial membrane potential, which in turn serves as an early signal in the apoptotic process. Thus, the decline of MMP and cardiolipin levels observed in our study supports a close link between the anti-cancer effect of curcuminoid analogues and mitochondrial dysfunction. There is mounting evidence that mitochondria are involved in the therapeutic properties of curcumin, which aligns with our study [31–36]. But most of the studies that involved ROS in the curcumin-induced mitochondrial pathway would report curcumin-induced mitochondrial dysfunction through ROS acting as the main apoptotic signal causing mitochondria damage or either through MMP dissipation that leads to ROS production inducing apoptosis, and thus the role of ROS would be confirmed through NAC action [37–40]. To our knowledge, this is the first demonstration that curcumin-related compounds, even after blocking ROS production, continue to induce apoptosis, suggesting that these curcuminoid analogues are able to exert their action with ROS-independent pathway and very few studies have been reported on this particular pathway [29, 30].

Caspases are inactive monomeric precursor enzymes (pro-caspase) that must be cleaved and dimerized for full activation and have critical roles in promoting the apoptosis pathway through activation of the caspase cascade [41]. The importance of caspase in inducing cell death was elucidated in our study as inhibition of caspase action through general caspase inhibitor reduced the apoptosis caused by all the research compounds. In addition, our study found that curcuminoid analogues and curcumin induced the decline in pro-caspase-9 and pro-caspase-9 expressions, indicating the cleavage of these caspases into active forms, suggesting the activation of the intrinsic apoptotic pathway. In the intrinsic apoptotic pathway, mitochondrial outer membrane permeabilization would likely occur following mitochondria damage. As a result, cytochrome c is released, and it plays a crucial role in activating caspase-9. The release of cytochrome c into the cytoplasm led to the formation of an apoptosome complex, composed of cytochrome c, the adaptor protein Apaf-1, and caspase-9. This complex, in turn, initiates the activation of caspase-3 [42, 43]. The involvement of the intrinsic pathway in curcuminoid analogues-induced apoptosis, including curcumin, was confirmed through the activation of caspase-9, as observed in the decline of pro-caspase-9 expression.

Furthermore, we also assessed the expression level of pro-caspase-8 to identify the ability of these analogues to induce the death-receptor apoptosis pathway. Our findings reveal that FLDP-5 and FLDP-8 curcuminoid analogues induced the early decline in pro-caspase-8 expression, indicating the cleavage of this caspase into its active form. These results strongly suggest that these compounds can initiate the extrinsic apoptotic pathway in LN-18 cells. In contrast, curcumin did not induce an early activation of caspase-8, indicating that this compound-induced apoptosis does not activate the death receptor pathway. Our results agree with studies that reported curcumin-induced apoptosis through only ROS-mediated mitochondrial pathways as indicated by mitochondrial damage followed by activation of caspase-9 and caspase-3 [44–46]. Moreover, through the time-point treatment of the pro-caspases activity in the immunoblotting results, we can see that caspase-8 is activated earlier than caspase-9 in both curcuminoid analogues treated cells suggesting that caspase-8 may mediate the processing of Bid to form tBid and trigger the intrinsic apoptotic pathway. This may happen due to the apoptosis-supporting role of cardiolipin after its externalisation into the outer membrane, which serves as a receptor and provides an activating platform to recruit tBid to the mitochondrial outer membrane, thus activating the intrinsic apoptotic pathway [47, 48].

miRNAs play a role in tumour pathogenesis by acting as oncogenes or tumour suppressor genes, making miRNAs excellent tools for cancer molecular diagnostics and targeted molecular therapy. Among cancer-related miRNAs, miRNA-21 is overexpressed in nearly every type of malignant tumour, including GBM and has been reported to be mediated in cancer-related processes [49–52]. Thus, this miRNA has gained attention as a target for inducing cell death in the therapy of cancer, as downregulation of this particular microRNA has been reported to modulate the apoptosis pathway through various signaling pathways such as PI3K/ Akt/NF-κB and PTEN/PI3K/AKT [53–55]. Our findings demonstrated that treatment of LN-18 cells with curcuminoid analogues (FLDP-5 and FLDP-8) and curcumin caused a significant dose-dependent suppression in the expression of miRNA-21 and thus may involve the cell death process of LN-18 cells. According to previous studies, treatment with curcumin in human U87 glioma cells was able to downregulate miRNA-21 expression by antisense oligonucleotides, inhibit glioma cell proliferation, and induce cell apoptosis through activation of caspase-3 and caspase-9 [56]. The study by Qiang and colleagues also found that curcumin modulates the miR-21/PTEN/Akt pathway in the human gastric cancer MGC-803 cell line. Curcumin elevated PTEN expression and down-regulated miRNA-21 levels in MGC-803 cells, resulting in cell apoptosis [57]. Therefore, additional thorough research should investigate the underlying mechanism of miRNA-21 suppression induced by these analogues, as it could provide a better understanding of their anti-cancer effects.

**Fig. 9:**
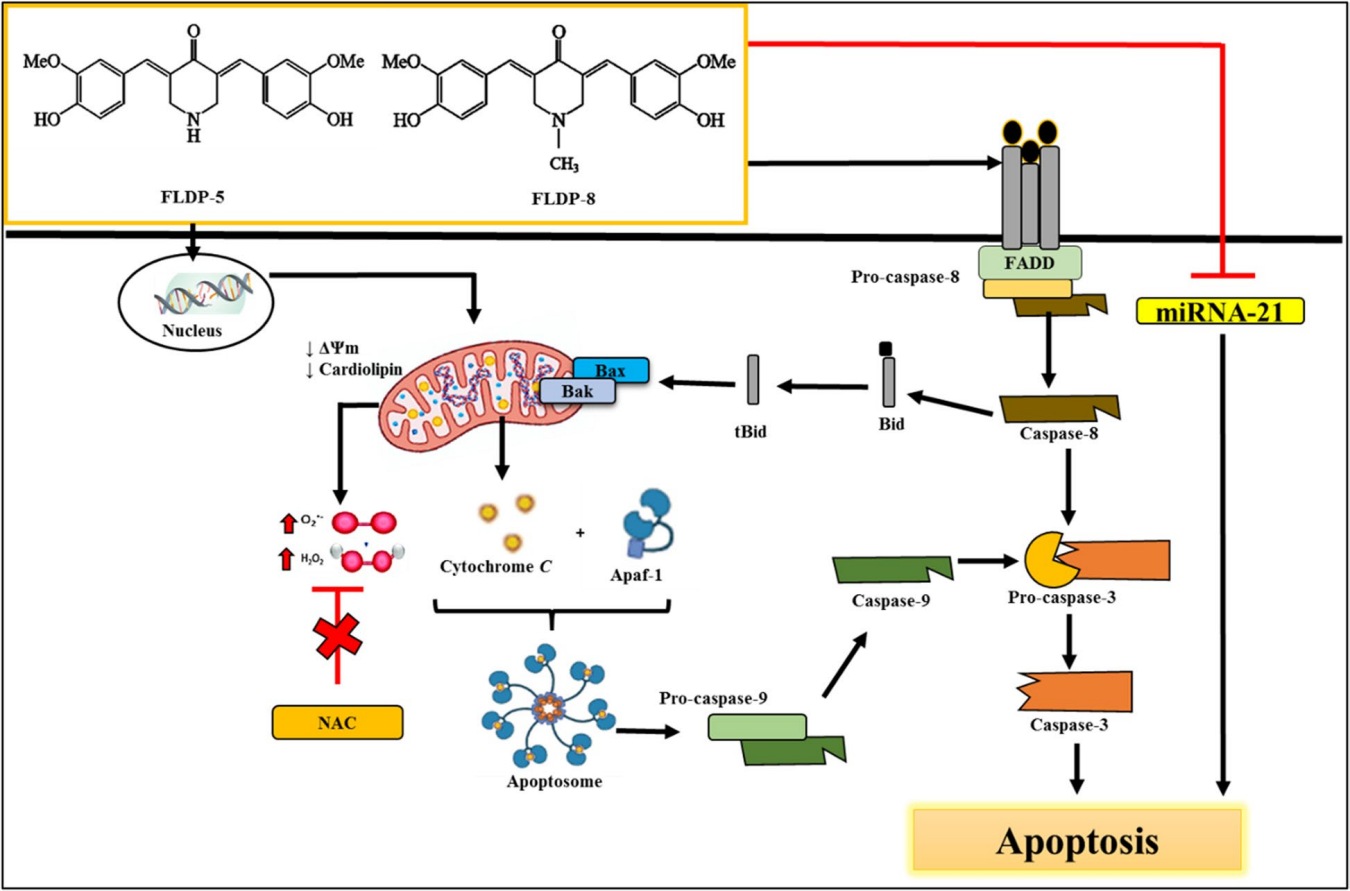
Schematic representation of curcuminoid analogues (FLDP-5 and FLDP-8)-induced apoptosis in LN-18 human GBM cells. The FLDP-5 and FLDP-8 curcuminoid analogues induce apoptosis in LN-18 cells via both intrinsic and extrinsic pathways. Through the cleavage of pro-caspase-8, both analogues induce an extrinsic apoptotic pathway. The active caspase-8 then cleaves the downstream effector pro-caspase-3 as well as the proapoptotic protein Bid, changing them to active forms. The resulting tBid then induces the release of mitochondrial proapoptotic components, potentially connecting the two pathways. The curcuminoid analogues FLDP-5 and FLDP-8 also induce the intrinsic apoptotic pathway. This pathway is activated by an early DNA damage that impacts the mitochondria, resulting in MMP and cardiolipin loss. This impact initiates the release of cytochrome c into the cytoplasm, ultimately forming an apoptosome with Apaf-1. This is followed by the cleavage of pro-caspase-9, which cleaves executor pro-caspase-3, the key player in the downstream events of apoptosis. Both curcuminoid analogues were also suggested to induce apoptosis by suppressing miRNA-21

**Fig. 10:**
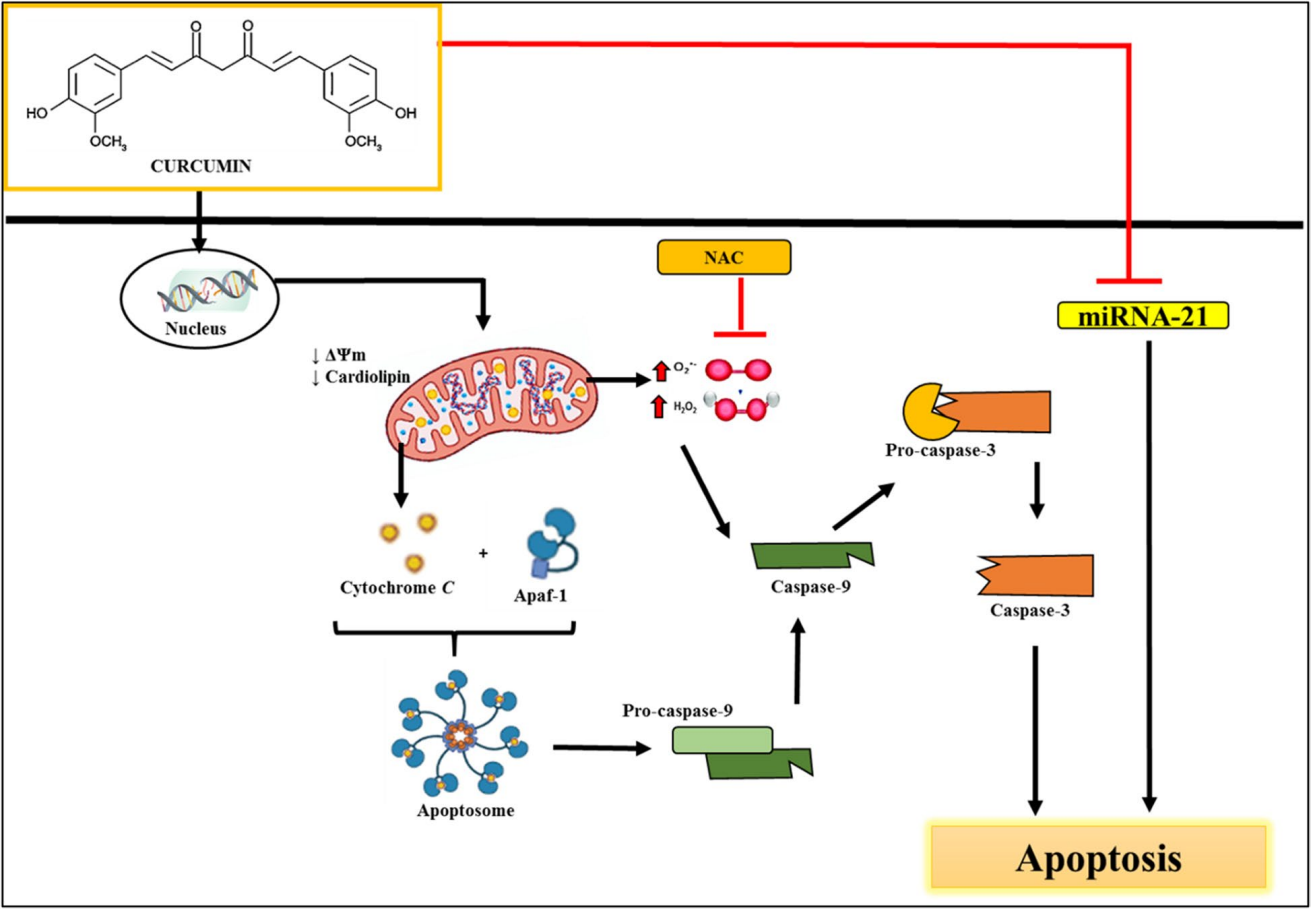
Schematic representation of curcumin-induced apoptosis in LN-18 human GBM cells. Curcumin induces intrinsic apoptosis by causing early DNA damage and disrupting mitochondrial function. This leads to MMP and cardiolipin loss, potentially prompting the release of cytochrome c into the cytoplasm. The release of cytochrome c leads to the cleavage of pro-caspase-9, changing it into an active form. This triggers the formation of an apoptosome complex, comprising cytochrome c, the adaptor protein Apaf-1, and active caspase-9. Subsequently, this complex cleaves and activates executor caspase-3, initiating apoptosis. NAC pretreatment inhibits curcumin-induced apoptosis, indicating the significant role of ROS in the cell death process of curcumin-treated LN-18 cells. Curcumin was also suggested to induce apoptosis through the suppression of miRNA-21

Overall, our findings elucidate the underlying mechanism of apoptosis in LN-18 cells treated with the novel compounds FLDP-5 and FLDP-8 curcuminoid analogues compared to curcumin. These findings demonstrated that these analogues possessed potent anti-cancer activity over its parent compound, curcumin, with slightly different action mechanisms in inducing LN-18 cell death, as summarized in the schematic representation in Figs. 9 and 10.

## Conclusion

In summary, we have shown that FLDP-5 and FLDP-8 curcuminoid analogues were able to exert their potential to be developed as anti-cancer agents through the suppression of miRNA-21 and caspase-dependent activation of the intrinsic mitochondrial-mediated and extrinsic apoptosis pathway in LN-18 human GBM cells, with higher potential that curcumin. A distinguished ROS-independent pathway induced by these analogues also may provide new insight into apoptosis-targeted therapy. Taken together, this study enhances our understanding of the molecular mechanism of activation-induced apoptosis in LN-18 cells. A more comprehensive investigation into the anti-cancer effects could significantly contribute to the development of curcumin-related compounds for apoptosis-targeted therapies in the future.

## Data Availability

All data generated or analyzed during this study are included in this published article. The data are available from the corresponding author upon request.
